# Cost Evaluation Analysis of Genetic Testing and Tailored Adjuvant Imatinib in Patients With Resected High-Risk GI Stromal Tumors: The Brazilian Perspective

**DOI:** 10.1200/GO.23.00070

**Published:** 2023-10-19

**Authors:** Bruna Bianca Lopes David, Pedro Nazareth Aguiar Junior, Matheus Costa e Silva, Rodrigo Dienstmann, Carlos Gil Ferreira, César Serrano

**Affiliations:** ^1^ Grupo Oncoclinicas, São Paulo, Brazil; ^2^ Division of Clinical Research and Technological Development, Brazilian National Cancer Institute, Rio de Janeiro, Brazil; ^3^ Hospital Vall d’Hebron, Barcelona, Spain

## Abstract

**PURPOSE:**

Mutations of the *KIT* gene are the molecular hallmark of most GI stromal tumors (GISTs). Imatinib has revolutionized GIST treatment. Adjuvant imatinib for 3 years is the standard of care for high-risk resected GIST. However, the GIST molecular biologic profile has found different responses to this approach. Despite this, genetic testing at diagnosis is not a routine and empirical adjuvant imatinib remains the rule. Barriers to genetic profiling include concerns about the cost and utility of testing. This analysis aims to determine whether targeted genetic testing reduces costs as an ancillary tool for a limited-resource scenario instead of adjuvant empirical imatinib in patients with resected high-risk GIST.

**METHODS:**

The cost evaluation analysis of molecular testing for GIST was based on the Cost of Preventing an Event (COPE), considering the Number Needed to Treat and the costs of each test compared with the cost of 3-year empirical adjuvant imatinib and real treatment costs (median number of cycles) from the public and private Brazilian Healthcare System's perspective. The analysis compared the costs of the molecular tests (broad next-generation sequencing [NGS], GS Infinity DNA/RNA assay, and targeted NGS: GS Focus GIST and the Fleury GIST Tumor DNA sequencing panel), costs of drug acquisition, considering discounts (imatinib mesylate and Glivec), and the costs of supportive care.

**RESULTS:**

In both scenarios, public and private, regardless of the use of imatinib or Glivec, tailoring adjuvant treatment reduced costs, irrespective of the number of cycles. The only exception was the combination of the broad NGS test and imatinib in the Public Healthcare System.

**CONCLUSION:**

The molecularly tailored adjuvant imatinib reduced costs considering the COPE of available NGS tests for both the public and private Brazilian health care systems.

## Teaser

In the Brazilian context, molecularly tailored adjuvant imatinib reduces costs over empirical treatment for GISTs.

## INTRODUCTION

GI stromal tumors (GISTs) are the most common malignant neoplasm of mesenchymal origin.^1^ GISTs predominantly originate in the stomach (60%) and small intestine (30%) although they can arise virtually at any portion of the GI tract.^1,2^ In the late 1990s, the understanding of the essential role of receptor tyrosine kinases KIT or PDGFRA in GIST oncogenesis together with the use of targeted agents such as first-line imatinib marked a new era in GIST therapeutics.^3,4^

**CONTEXT**

**Key Objective**
To demonstrate the cost evaluation analysis of molecularly tailored adjuvant imatinib for resected high-risk GI stromal tumors (GISTs) when compared with empirical imatinib for 3 years in the reality of public and private Brazilian health systems, using different next-generation sequencing tests.
**Knowledge Generated**
In the Brazilian context, targeted genetic testing reduces costs as an ancillary tool instead of adjuvant empirical imatinib in patients with resected high-risk GIST.
**Relevance**
The molecular heterogeneity of GISTs is well known, but molecular test is not a routine in the daily basis, and adjuvant imatinib for 3 years is the standard of care for high-risk resected GIST. Systematic testing could spare patients from unnecessary toxicity, better direct resources, and open an opportunity to build biobanks in a rare entity and limited-resource scenario.

Further research undertaken thereafter demonstrated that the GIST molecular profile is heterogeneous and has an impact on clinical management and outcomes of patients with GIST. Approximately 70% of the tumors harbor *KIT* mutations that arise more frequently in exons 11 (67%) and 9 (10%-15%), whereas exons 13 and 17 account for <1% of the patients. *PDGFRA* mutations in exons 12, 14, and 18 occur in approximately 15% of all patients with GIST.^5^ There is also a proportion of GIST (10%-15%) that lacks mutations in *KIT* and *PDGFRA* (the so-called wild-type [WT] tumors). These tumors harbor other driver alterations involving *RAS* pathway activation, such as *RAS*, *BRAF*, and *NF1*; succinate dehydrogenase complex deficiency; and *NTRK3* or *FGFR1* fusions. The latter molecular subgroups differ from *KIT*- and *PDGFRA*-mutant GISTs in terms of clinical features, patient outcomes, and imatinib responsiveness.^6-9^

On the basis of data from the SSGXVIII/AIO randomized phase III trial,^10,11^ adjuvant treatment with imatinib 400 mg once daily for at least 3 years is recommended for patients with resected high-risk GIST, regardless of mutation status.^12-14^ Several prognostic criteria have been proposed, and variables such as tumor size, mitotic rate, primary tumor site, and tumor rupture are currently widely established in the daily clinical practice to predict the risk of recurrence and/or metastases.^2,15-17^ Estimating the risk of relapse after tumor resection is therefore important to define the specific patients who will benefit from adjuvant treatment.

Remarkably, the value of molecular testing for risk stratification after primary tumor resection is largely unknown. A wealth of evidence supports that certain genotypes, such as WT GISTs and *PDGFRA* D842V–mutant tumors, are, respectively, independent of KIT/PDGFRA oncogenic signaling or resistant to imatinib. Consequently, the potential benefit of adjuvant imatinib in these genotypes is uncertain and, likely, null, given the little to no activity shown by imatinib in the metastatic setting.^17^ On the basis of this evidence, it is recommended that patients with WT GIST and those harboring the multiresistant *PDGFRA* D842V mutation should not receive adjuvant therapy.^17^ For individuals whose tumors harbor *KIT* exon 9 mutations, higher-dose imatinib therapy may be preferred,^18,19^ if tolerated, but it is not a consensus in the adjuvant setting.^12-14,20^ Despite the accuracy of KIT/PDGFRA genotyping in predicting the activity of imatinib in GIST, from 15% to 33% of patients undergo genetic testing at diagnosis across various countries and socioeconomic scenarios.^21-24^ Consequently, imatinib 400 mg once daily is commonly delivered as the standard adjuvant therapy for all high-risk localized GISTs without considering the tumor molecular profile.

The cost-effectiveness of imatinib in the adjuvant setting seems well-established. Several analyses have demonstrated that 3 years of adjuvant imatinib is more cost-effective for treating localized primary GISTs than surgery alone or just 1 year of adjuvant therapy. Incremental prescription drug costs have been shown to be partially offset by the reduction in costs associated with delayed GIST recurrence. The appropriate selection of patients with high risk of recurrence and sensitive mutations is a cost-saving approach.^25,26^

Although tyrosine kinase inhibitors (TKIs) like imatinib are described as well tolerated with manageable side effects, almost all patients treated with this TKI experience at least one adverse event,^27^ which may affect patients' quality of life.^28^ This, in turn, can increase not only the final treatment costs but also monitoring costs as more visits and diagnostic tests are eventually required.

In this sense, the systematic use of genetic testing to tailor the adjuvant treatment could help spare patients from toxicity and optimize costs, especially in the context of scarce resources. Health care stakeholders usually have concerns regarding the cost and utility of genomic profiling, which constitutes a barrier to access to such technology. In this study, we assessed whether molecular testing is a cost reduction ancillary tool to guide adjuvant imatinib in patients with surgically resected high-risk GIST in the Brazilian health care system environment.

## METHODS

This study assessed the cost reduction power of molecular testing for GIST on the basis of the Cost of Preventing an Event (COPE), which considers the Number Needed to Treat (or to Test, in the case of the present study [number needed to treat [NNT]]) and the costs of each test compared with the cost of 3-year empirical adjuvant imatinib. The NNT is the inverse of the absolute risk reduction, defined as the risk of adjuvant treatment according to each strategy arm (universal adjuvant treatment versus molecular-guided therapy). The analysis was based on projections of both, the Brazilian Public Healthcare System (SUS) and the Private Healthcare System scenarios.

### Cost Estimation

The economic analysis adopted the SUS and the Brazilian Private Healthcare System's perspective. The target population includes patients with resected high-risk GIST, candidates to receive 3 years of adjuvant imatinib. The molecular testing costs were included as the direct cost of all tests available in Brazil: broad next-generation sequencing (NGS) with FoundationOne CDx or GS Infinity DNA/RNA assay (local developed test from Oncoclínicas Precision Medicine that covers 463 genes) and targeted NGS with GS Focus GIST (Oncoclínicas Precision Medicine test that covers *KIT* and *PDGFRA*) or the comparable Fleury GIST Tumor DNA sequencing panel.

The treatment cost estimation encompasses direct costs such as imatinib acquisition, management, and supportive costs for adverse events management per person.

The evaluated pharmacologic products were imatinib mesylate (generic) and Glivec (Novartis), and their costs came from Medicines Market Regulation Chamber (CMED). The Maximum Price to the Consumer (PMC) was considered for the Brazilian Private Healthcare System, and the Maximum Sale Price to the Government (PMVG) was used for the Brazilian Public Healthcare System. PMC was assessed on September 2022 and PMVG on May 2022. The time on treatment was evaluated considering two scenarios: planned treatment duration and estimation of real treatment duration, on the basis of data from the SSGXVIII/AIO phase III trial comparing 3 years versus 1 year of adjuvant Imatinib. Therefore, planned treatment duration was defined as 36 months, whereas median treatment time was estimated assessing the disease-free survival (DFS) curve and the percentage of discontinuation for reasons other than progressive disease (PD). The resulting ratio of the Kaplan-Meyer Curve was collected and pondered with the discontinuation ratio observed in correlated studies.^10^ The AUC for the average progression was calculated by a trapezoidal integration through the jackknife (or leave-one-out) resampling technique. The median time to progression obtained was used to estimate the median number of cycles. The R programming language and environment, and the flux package were used to perform these evaluations.

The costs of supportive care for adverse events were calculated using the formula for correcting the purchasing power parity of Brazil's gross domestic product per capita according to the World Bank 2021. The applied exchange rate was Brazilian Reais (R$) 5.20. The authors also considered a micro-costing technique to estimate the adverse events supporting costs. In this analysis, for the Private Healthcare System, the costs of each procedure were retrieved from Brazilian Hierarchical Classification of Medical Procedures from the Brazilian Medical Association published in 2018 and updated in 2021 (CBHPM-AMB 2018 Porte 2021). The costs for the SUS were estimated from the Procedure Table Management System (SIGTAP). The most common adverse events related to imatinib were included, as follows: anemia, nausea, leukopenia, diarrhea, fatigue, and their frequency on the basis of literature data.^21^

Monitoring costs for the adjuvant period were calculated considering a monthly periodicity during TKI and 3-month schedules during follow-up. It was translated in a difference of 24 more visits and blood tests for the universal adjuvant choice than molecular-guided, during a 3-year period. Laboratory tests included hemogram and kidney and liver function. Monitoring costs were also retrieved from CBHPM-AMB 2018, Porte 2021 for the private health system and from SIGTAP for the SUS. Imaging scans were not considered since patients under adjuvant treatment or just clinical follow-up are supposed to have the same schedule of examinations.

### COPE Analysis

The authors considered the risk of adjuvant treatment as 100% among patients without genomic testing and 90% among patients with molecular-guided treatment (10% of GISTs as wild-type). The risk of adjuvant treatment among tested patients was corrected by the sensitivity of the test for imatinib-resistant alterations and the success rate of the test (eg, NGS failure because of poor quality of the DNA/RNA). The formula used was 0.90 + (0.10 × [1 – Test Sensitivity × Test Success Rate]). The absolute risk reduction was obtained by subtracting one from this result and then the NNT, which values were rounded up to the next whole number.

COPE was obtained from the cost of each test multiplied by the NNT to prevent one adjuvant treatment. This cost was compared with planned treatment costs and real treatment costs. In cases that COPE was lower than that of adjuvant treatment, the molecular testing strategy was considered as cost-effective. In cases where COPE was higher, the molecular testing strategy was considered as not cost-effective.

To assess the robustness of the findings, possible variations in the reference costs were considered. The PMC and PMVG rates can differ from real costs as there are commercial deals for each health care provider. In this sense, an estimated discount was applied for each drug (imatinib or Glivec) to promote a more competitive and realistic comparison.

## RESULTS

### Cost Estimation

Testing costs, sensitivity, test success rate, and NNT for each modality of the ancillary test are shown in Table 1. The median number of imatinib cycles was estimated to be 26. Each cycle was calculated in a monthly schedule. On the basis of this, the costs of the drugs in the public and private scenario by time comparing 26 cycles with the planned treatment period of 36 cycles are listed in Table 2.

**TABLE 1:**
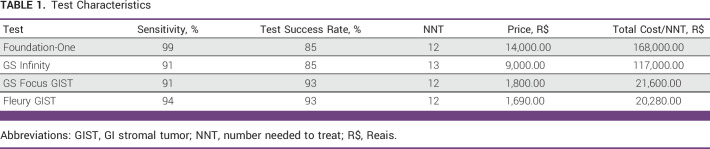
Test Characteristics

**TABLE 2:**
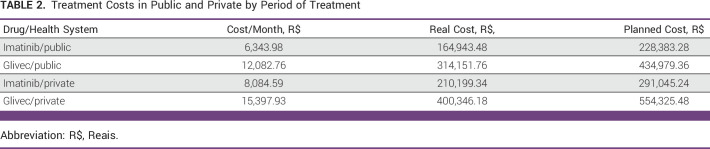
Treatment Costs in Public and Private by Period of Treatment

Prices for the management of the most common adverse events, including anemia, leukopenia, fatigue, nausea, and diarrhea, and the monitoring costs, also in the public and private practice, are listed in Table 3.

**TABLE 3:**
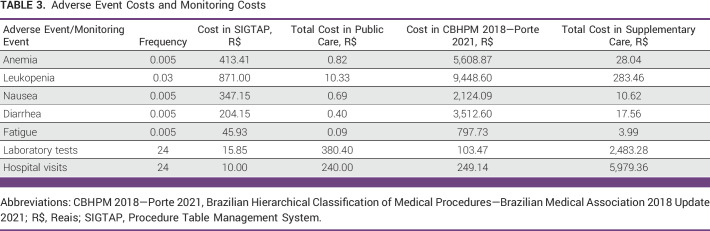
Adverse Event Costs and Monitoring Costs

### COPE for Each Test Versus Empirical Adjuvant Imatinib Comparison

**FIG 1:**
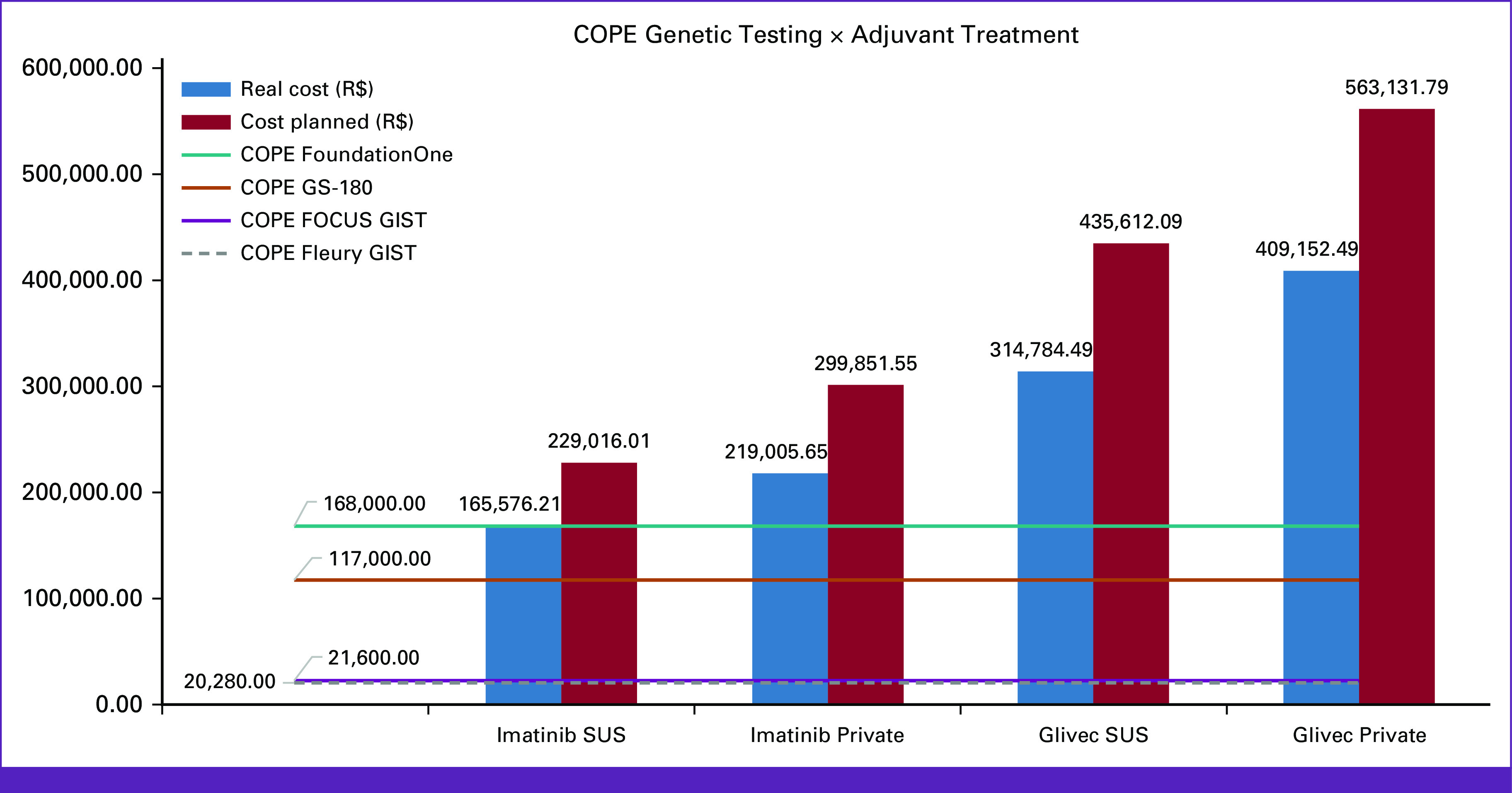
COPE genetic testing compared with adjuvant treatment in the private and public setting. Values in Reais (R$); COPE, cost of preventing an event; GIST, GI stromal tumor; SUS, Sistema Unico de Saude—Brazilian Public Health System.

COPE results for each test were compared with the total cost of the adjuvant treatment, which included drug prices, adverse events, and monitoring costs, for the public and private context. In both scenarios, regardless of the use of imatinib or Glivec, the COPE of genomic testing before adjuvant treatment was cheaper, irrespective of the number of cycles (planned × median). The only exception was the combination of the FoundationOne CDx test and imatinib in the Public Healthcare System, where the COPE of the test was more expensive than the universal use of the drug. The GS Focus GIST and GS Fleury GIST were the cheapest modalities, with a COPE of R$ 21,600.00 and R$ 20,280.00 respectively; FoundationOne CDx costs were R$ 168,000.00, and GS Infinity R$ 117,000.00. Figure 1 summarizes the comparison between tests and systemic treatment, on the basis of a 3-year period (cost planned) and the 26-cycle duration (real cost).

When considering drug price discounts, usually practiced in the market, two KIT- and PDGFRA-focused tests, GS Focus GIST and Fleury GIST, were better choices even with a price reduction of around 90% in the established values. These results are also maintained when applied to the Private Healthcare System, with discounts of 97% in the Glivec price being necessary to overcome the COPE of GS Focus GIST or Fleury GIST.

## DISCUSSION

In the daily clinical practice, the gold standard treatment for surgically resected high-risk GIST is at least 3 years of adjuvant imatinib on the basis of data from the Scandinavian Sarcoma Group (SSG) XVIII trial.^10,11^ This randomized phase III clinical trial demonstrated a significant improvement in both DFS and overall survival (OS) when compared with 1 year of treatment. This benefit persisted over time, as observed in a later report with a follow-up of 119 months.^11^ However, it is known that the molecular heterogeneity of GISTs confers variable patterns of responsiveness to imatinib. Thus, although all patients with high-risk GIST were included in the SSG XVIII pivotal clinical trial, there is broad consensus nowadays from sarcoma expert oncologists to not offer systemic adjuvant therapy to patients whose tumors are classified as *KIT* and *PDGFRA* WT nor to *PDGFRA* D842V–mutant GIST.^12-14^ Taken together, the total amount of primary resistant patients can reach up to 15%-20% of all cases.^5-9^

**FIG A1:**
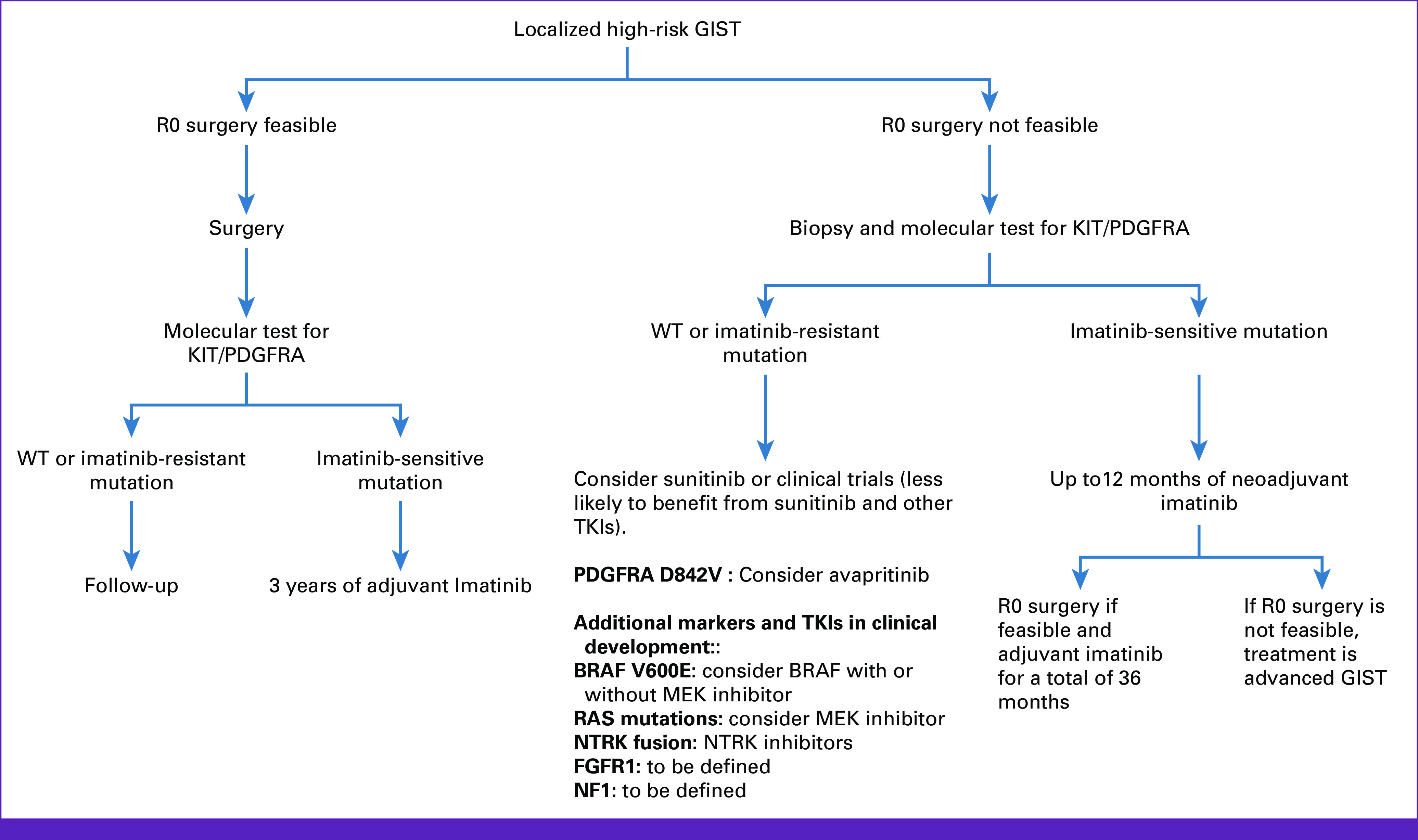
Algorithm for managing localized high-risk GIST. FUP, follow-up; GIST, GI stromal tumor; TKI, tyrosine kinase inhibitor; WT, wild type.

Despite important advances in GIST molecular profiling, the percentage of testing before adjuvant treatment is extremely low, from 15% to 33%.^21-24^ Barriers to adopt testing may involve concerns about the cost and utility. In the metastatic scenario, some data suggest that using genetic testing to match treatment of *KIT* variations to imatinib is a cost-effective approach compared with empirical imatinib.^29^ It is of great value when considering that 30% of newly diagnosed patients have localized disease, of which 40% are considered at high risk and could be candidates for the adjuvant treatment.^21^ Because of this fact, both European Society of Medical Oncology, National Comprehensive Cancer Network, and SELNET guidelines advocate that mutational analysis should always be considered standard practice, especially for patients who are candidates for systemic therapy.^12-14^ The test has not only a predictive value for sensitivity to molecular-targeted therapy but also a prognostic relevance. Appendix Figure A1 illustrates a proposed flowchart for managing high-risk localized GIST.

Tests’ performance is a fundamental topic when it comes to recommend such an important adjuvant intervention. The high proportion of mutant GISTs missed by the Sanger method reinforces the need to offer tumor NGS up front.^30^ In the exposed analysis, all the NGS tests evaluated are certified and the failure rate varies from 7% to 15%, depending on the requirements for presequencing quality metrics. Still, these values are considered acceptable, and the four tests evaluated have high levels of accuracy. The available diagnostic panels herein discussed vary by read breadth and depth. The least costly of these involve focused gene panels, whereas whole-exome diagnostics are more expensive. In the context of limited resources, promoting the local technology and more focused panels could be cost saving.

Regarding the health economics, interventions with higher NNT will, in theory, waste a greater number of resources to obtain a successful case for treatment. Thus, COPE can be a tool for cost evaluation analysis, especially for curative procedures. In this study, tailoring adjuvant imatinib in resected high-risk patients on the basis of the genomic profiling reduced costs in all base case scenarios. It is important to emphasize that the most focused tests, encompassing just KIT and PDGFRA analyses, minimized costs even when simulations of market discounts for imatinib were as high as 90% of the reference cost.

The optimal duration of adjuvant imatinib is also an open question. Three large, randomized trials have addressed this issue by evaluating mixed patient groups and treatment times ranging from 1 to 3 years. But for how long? The longest study, 3 years, showed an OS gain not seen in those who used the medication for 1 or 2 years.^10,25,26,31^ This may indicate that the impact of adjuvant imatinib on OS would be related to longer duration of use. The use of a surrogate end point, the imatinib failure-free survival, was not validated, but its use seems interesting as adjuvant and first-line therapies are the same. In the case of GISTs, secondary resistance to imatinib is reflected in limited benefit to further therapeutic lines, directly affecting the OS.^26,31^ Adjuvant imatinib for 3 years is still the standard for high-risk, mutation-sensitive GISTs. Data evaluating the safety and benefit of increasing this time are not conclusive (ClinicalTrials.gov identifiers: NCT02413736, NCT02260505).^32^ If a new consensus of care with longer periods of treatment becomes the standard, the costs will also rise, reassuring the need for a molecular tailored decision to avoid waste of resources and, clearly, to spare patients from toxicity. Moreover, the present analysis does not include direct nonmedical costs such as the costs of travel and food and opportunity cost of caregivers, which is higher during the adjuvant period.

For resectable GIST, 3-year adjuvant imatinib therapy represents a cost-effective treatment option. However, it is important to highlight that most of the studies were conducted in high-income and developed countries, including European and American ones. The exception is one study where the adjuvant therapy was likely to be cost-effective, but because of the current imatinib price in the authors’ country, this could not be proved considering the context.^31^

Quality of life is also an increasingly recognized parameter to consider during imatinib adjuvant treatment, given its long duration. Although the discontinuation rate because of adverse events in imatinib adjuvant trials fluctuates between 12% and 16%,^10,32-35^ almost half of the patients stopped the drug early in a phase II trial, and in the phase III studies, nearly one quarter of patients do so. This suggests that, even with a drug that is well tolerated and has relatively mild side effects, it is still hard to convince patients to stay on this drug for a longer duration. In the current analysis, the median number of imatinib cycles was based on the DFS curve and the percentage of discontinuation for reasons other than PD from the SSGXVIII/AIO phase III trial. The final results showed an estimated number of 26 monthly cycles in the whole study population, which reassures that drug discontinuation is not a banal event.

This analysis has some limitations since it was based on projections and not on a population database. However, it brings interesting data about an issue not yet explored in the Brazilian scenario, where resources are scarce and rare cancers suffer from deprioritization. It is important to emphasize that Brazilian Healthcare is marked by a dualism, whereby 25% of the population has access to private insurance and the remaining 75% is covered by the SUS. Both health care systems face budgetary, governance, and organizational challenges, which affects their sustainability and capability to provide equitable health care.^36^ The data presented here suggest that, in the public and private scenario, genetic testing in patients with resected high-risk GIST could be an efficient way of optimizing resource allocation.

In conclusion, the molecularly guided indication of adjuvant imatinib in patients with surgically resected high-risk GIST reduced costs considering the COPE of NGS available tests. This result was observed for both the public and private health care systems, even considering drug acquisition discounts.
